# Hepatitis C Virus Induces Regulatory T Cells by Naturally Occurring Viral Variants to Suppress T Cell Responses

**DOI:** 10.1155/2011/806061

**Published:** 2010-12-06

**Authors:** Matthew F. Cusick, Jennifer J. Schiller, Joan C. Gill, David D. Eckels

**Affiliations:** ^1^Division of Histocompatibility and Immunogenetics, Department of Pathology, University of Utah School of Medicine, Salt Lake City, UT 84132, USA; ^2^Blood Research Institute, BloodCenter of Wisconsin, Milwaukee, WI 53226, USA

## Abstract

Regulatory T cell markers are increased in chronically infected individuals with the hepatitis C virus (HCV), but to date, the induction and maintenance of Tregs in HCV infection has not been clearly defined. In this paper, we demonstrate that naturally occurring viral variants suppress T cell responses to cognate NS3_358-375_ in an antigen-specific manner. Of four archetypal variants, S370P induced regulatory T cell markers in comparison to NS3_358-375_-stimulated CD4 T cells. Further, the addition of variant-specific CD4 T cells back into a polyclonal culture in a dose-dependent manner inhibited the T cell response. These results suggest that HCV is able to induce antigen-specific regulatory T cells to suppress the antiviral T cell response in an antigen-specific manner, thus contributing to a niche within the host that could be conducive to HCV persistence.

## 1. Introduction

Hepatitis C Virus (HCV) may evade the immune response or impart a specific tolerance to itself to ensure its survival in over 80% of infected individuals through mechanisms such as, but not excusive to, viral escape, T-cell energy, and induction of regulatory T cells (Treg). 

Recent studies on hepatitis C virus (HCV) have described an increase in Treg markers in cohorts of chronically infected patients when compared to resolved and noninfected individuals, possibly leading to viral persistence [1–7]. Although these studies suggest a correlation between Treg cell numbers and HCV clearance, it has not been determined if Tregs are induced in an antigen-specific manner or upregulated to inhibit immunopathological damage associated with a chronic infection. 

There are two main subsets of Tregs: (I) thymically selected natural Tregs (nTreg), which are phenotypically defined as CD4^+^ CD25^hi^ Foxp3^+^, and (II) “inducible” Treg cells, activated in the periphery, termed either Tr1 or Th3 defined as secreting IL-10, TGF*β*, and possibly IL-4 [8, 9]. A variety of markers are available to define Tregs, but the most generally accepted marker is the expression of Forkhead Box P3 (Foxp3). This expression positively correlates with the development of Treg cells that have the capacity to suppress the in vitro and in vivo proliferation and function of effector T cells [10–14]. Recent studies have found a correlation between *α*-chain of IL7R (CD127) and Treg cells [15]. Golden-Mason et al. also found a correlation between CD127 expression and the virological outcome of acute HCV suggesting a relationship between HCV persistence and an increase in Treg cells [16].

Previous work in our laboratory demonstrated a functional induction of IL-10 in CD4 T cells in chronic HCV subjects, indicative of inducible Treg cells, as opposed to resolved HCV subjects which secreted IL-2 and IFN*γ* [17, 18]. Further, screening for immunodominant epitopes in one chronic HCV subject, using an array of synthetic peptides, found an IFN*γ* and IL-2 producing epitope NS3_358–375_ showing a distinct cytokine profile in contrast to the rNS3 protein-stimulated PBMC [19]. In a longitudinal study tracking viral variants in a chronic HCV subject, we identified viral variants consistent with selective immune pressure [20]. One variant, S370P, was noted to be stable for over 2 years indicating selection and fixation of this HCV viral isolate [20, 21]. Simple escape and redirection of the immune response does not explain, however, the maintenance of an abundant population of wild-type HCV sequences in infected patient's even years into an ongoing infection. This paradox is that viral genomes persist in the presence of T cells, which should be able to specifically recognize and help to clear virus infected cells, and suggests there may be another level of immunoregulation that is modulated by the viral infection [22–26]. Based on these observations, we hypothesize that a Treg population specifically suppresses the response of the effector T cells to the HCV antigens, and this Treg-mediated suppressive activity is induced by naturally occurring viral variants that accumulate mutations in an important viral epitope recognized by helper T cells.

In the present study, we evaluated the role of naturally occurring viral variants in the suppression of T cell responses to cognate NS3_358–375_ in vitro. Of four archetypal variants, the S370P variant induced regulatory T cell markers in comparison to NS3_358–375_-stimulated CD4 T cells. Further, adding variant specific CD4 T cells back into a polyclonal culture, in a dose-dependent manner, inhibited the T cell response to cognate NS3_358–375_. These results suggest that HCV may be able to induce regulatory T cells to suppress the antiviral T cell response in an antigen-specific manner, potentially creating a niche within the host that could be conducive to HCV persistence.

## 2. Materials and Methods

### 2.1. Patients

Blood was collected in acid citrate dextrose, processed for PBMC isolation over lymphocyte separation medium, and preserved in liquid nitrogen, as previously described [27]. DNA was isolated from whole blood and sent for HLA typing at the University of Utah (Table 1), and the lymphocytes were incubated with various concentrations of rNS3 to test for T cell responses. Quantitative RT-PCR and HCV genotyping on all serum samples were sent to ARUP laboratories (Salt Lake City, UT). All chronic HCV subjects used in this study are genotype 1a (Table 1). If the subjects had no detectable viral load, the samples were screened for HCV antibodies by recombinant immunoblot assay (RIBA) carried out at ARUP laboratories. These studies have been reviewed and approved by University of Utah Institutional Review Board and the Medical College of Wisconsin Institutional Review Board.

### 2.2. Cell Culture and Media

Culture of PBMC was in RPMI 1640 tissue-culture medium (BioWhittaker, Walkersville, ME) supplemented with 25 mM HEPES, 2 mM L-glutamine, 100 U/ml penicillin, 100 ug/ml streptomycin, 1 mM sodium pyruvate, 5 ug/ml gentamycin (all from Mediatech Cellgro, Herndon, VA), 10 U/ml heparin sodium (Fisher Scientific, Pittsburgh, PA), and 10% pure human serum (Atlanta Biologicals, Lawrenceville, GA). Cells were cultured in a 37°C, 5% CO_2_ incubator.

### 2.3. Synthetic Peptides and Protein

In vitro, PBMCs were stimulated with synthetic peptides representing one human leukocyte antigen DR15 (HLA-DR15) restricted epitope surrounding HCV NS3 amino acids 358–375 (aa 1384–1401 of the HCV polyprotein). The three single amino acid variants were identified in a chronic HCV patient (P.B3019), and recombinant nonstructural protein 3 (rNS3) protein was purified as previously described [17, 28]. Recombinant H3 (A/Phillipines/1992) and H5 (A/Vietnam/2004) were obtained from Protein Sciences. Peptides were synthesized using Fmoc chemistry and HPLC purified, dissolved in a small amount of DMSO, and then adjusted to 1 mg/ml stock solutions in RPMI 1640. Peptide sequences were as follows: wild-type 358–375, VIKGGRHLIFCHSKKKCD; variant H369R, VIKGGRHLIFCRSKKKCD; variant S370P, VIKGGRHLIFCHPKKKCD; variant K371E, VIKGGRHLIFCHSEKKCD.

### 2.4. T-Cell Proliferation Assay

To measure proliferative responses of PBMC following stimulation with wild-type peptide NS3_358–375_ and several variants, 1 × 10^5^ PBMC were plated in round-bottom 96—[29] incubated at 37°C, 5% CO_2_ for either 4 or 6 days, as indicated, pulsed overnight with 1 *μ*Ci/well of titrated thymidine (^3^H-TdR), and harvested onto glass fiber filters for measurement of radiolabel incorporation by gas scintillation spectroscopy. Results are presented as the mean ± SEM of at least triplicate cultures (typically 6 wells/sample were analyzed), and samples were compared using Student's unpaired *t*-test. Data were considered significant at *P* < .05. Proliferation data was transformed using a previously described algorithm: ${\log\;}_{10}\;\Delta \text{cpm}={\log\;}_{10}\;\left[{\overset{̅}{X}}_{\exp\;}-{\overset{̅}{X}}_{\exp\;}\right]$ [29].

### 2.5. Foxp3 Staining

PBMC were analyzed by flow cytometry to evaluate the frequency of Foxp3 in an expanding CD4^+^ T cell population when stimulated with various antigens in both HCV chronic and resolved subjects. Carboxyfluorescein succinimidyl ester (CFSE) staining protocol was adapted from Quah et al. [30]. Briefly, 0.5 *μ*M CFSE was added to PBMC in complete media + 10% PHS for 5 min. at 37°C, washed 3 times, and stimulated with the appropriate antigen(s) for 7 days. Cultures were then stained with CD4-Pacific Blue, CD8-Amcyan, CD25-APC, and CD127-percp-cy5.5 (BD Pharmingen, San Diego, CA) for 20 min. at 4°C and then washed 2x with stain buffer (BD Pharmingen, San Diego, CA). Using eBioscience Foxp3 staining kit, the cells were fixed and permeabilized for 1 hr at 4°C and then washed 2x in permeabilization buffer. Normal rat serum was added (2 *μ*l/100 *μ*l) for 15 minutes and then stained with Foxp3-PE (eBioscience, San Diego, CA) for 1 hr at 4°C, washed 2x with stain buffer, and analyzed on a BD FACS Canto II. To account for fluorescence spill over and nonspecific staining, we performed fluorescence-minus-one (FMO) with isotype control. More specifically, FMO controls contain all of the antibody conjugates used in the experiment except one, with the addition of the appropriate isotype control of the fluorochrome initially excluded. This was performed for each fluorochrome and each unique culture condition. For PH1127, the addition of IL-10 was added 3 hours after the appropriate antigen was added to culture at 2 ng/ml. Results were compared using Student's *t*-test. Flow cytometry data analysis was performed using Flow Jo software (Tree Star).

### 2.6. Magnetic Cell Sorting

To deplete CD4^+^CD25^+^ Treg cells from the PBMC, magnetically labeled microbeads were used and selected for using an autoMACS Separator (Miltenyi Biotec, Auburn, CA). First, non-CD4^+^ cells were labeled by incubation with a cocktail of biotin-labeled antihuman antibodies against CD8, CD14, CD16, CD19, CD36, CD56, CD123, TCR*γ*/*δ*, and glycophorin A, followed by addition of antibiotin microbeads, as recommended by the manufacturer of the CD4^+^CD25^+^ regulatory T cell isolation kit (Miltenyi Biotec). The non-CD4^+^ cells were then depleted with the autoMACS separator, resuspended in culture media, and set aside. CD25 microbeads were added to the remaining CD4^+^ cell pool and positively selected with the autoMACS. The resulting CD25-depleted CD4^+^ cells were pooled with the non-CD4^+^ cells and cultured with peptides as described above for proliferation assays.

### 2.7. Tetramer Staining

All tetramers were obtained from the NIH tetramer facility at Emory University. PBMC were stained immediately following thawing and washing. The CD4^+^T cells specific for NS3_358_ were amplified by stimulating PBMC from PB3019 with 1 *μ*M of cognate peptide and incubate the cells at 37°C, 5% CO_2_ for 7 days. The cells were then sorted at the University of Utah cell-sorting core facility. PBMCs were stained with CD4-APC (BD bioscience) and sorted under sterile conditions by gating on CD4^+^ CFSE^low^cells. The sorted cells were expanded with CD3/CD28 Dynal beads (Invitrogen) and 10 U/ml of rIL-2 (BD Bioscience) at 37°C, 5% CO_2_. Prior to staining with either wild-type 358–375-Phycoerythrin (PE), or variant H369R-Allophycocyanin (APC), variant S370P-APC, or variant K371E-APC tetramers, the CD3/CD28 beads were magnetically removed from the cultures, and the cells were stained with 2 *μ*g/ml of tetramer at 37°C for 1 hr in complete media plus 10% PHS. Extracellular surface staining was performed by adding 7-AAD cell viability probe, CD4-pacific blue (BD bioscience), CD3-Amcyan (BD bioscience), and CD8-FITC (eBioscience). Negative controls consisted of staining cells with nonspecific peptide, CLIP-DR15 tetramer, labeled with either -PE label or -APC, respectively, which were performed with each experiment and noninfected individuals.

### 2.8. Tetramer Depletion and Add Back Assays

Tetramer staining (10 *μ*g/ml) with the wild-type 358–375-Phycoerythrin (PE), variant H369R-Allophycocyanin (APC), variant S370P-APC, and variant K371E-APC were incubated with PBMC (1 × 10^6^ cells/50 *μ*l) in a 96 round well bottom plate for 90 min at RT temperature in the dark. The cells were washed, and MACS beads specific for either anti-APC or anti-PE were incubated with their respective tetramer for 15 minutes at 4°C. The cells were washed, and then applied to MS MACS column and tetramer depleted. Tetramer-positive cells were then collected and washed. Next, the cells were washed 2× in complete media + 10% PHS and then stimulated with the appropriate antigens. In the appropriate cultures, tetramer-positive cells were added to cultures in a dose-dependent manner based on volume. A CLIP-loaded tetramer for both PE and APC fluorochromes were used as a control for nonspecific staining.

## 3. Results

### 3.1. Foxp3 Expression in Chronic and Resolved HCV Subjects

**Figure 1:**
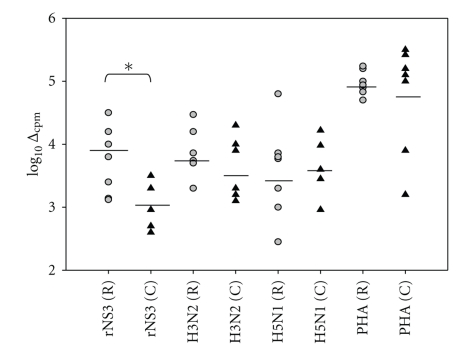
Resolved HCV subjects have a significantly higher T cell response to rNS3 than chronic HCV subjects. PBMCs from both resolved subjects (gray circle) (*n* = 8) and chronic (black triangle) (*n* = 8) subjects (subjects used in Figure 1 are ∗ in Table 1) were individually incubated with rNS3 (1 *μ*g/ml) and H3 (3 *μ*g/ml), H5 (3 *μ*g/ml), and PHA (2 *μ*g/ml) for 7 days. All subjects were screened in the same proliferation assay. As described in the results, the algorithm: log_10 _Δ_max_ was used to transform the data [29]. The *P* < .05* as determined by Student's *t*-test.

**Figure 2:**
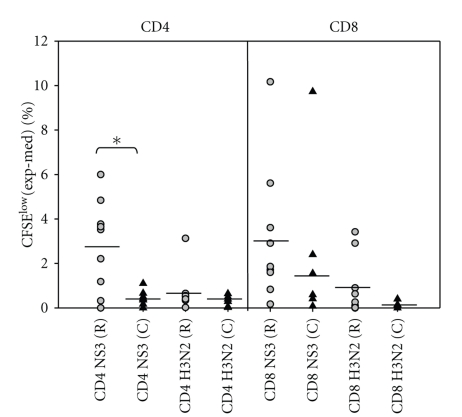
HCV subjects have an attenuated CD4^+^ T cell response to rNS3. HCV chronic subjects (black triangle) (*n* = 7) (Table 2) had a significantly lower CD4 CFSE^low^ response to rNS3 (1 *μ*g/ml) in comparison to resolved subjects (gray circle) (*n* = 9) (Table 2). There was no difference in the H3 (1 *μ*g/ml) response between groups. The *P* < .05* as determined by Student's *t*-test.

To compare chronic (*n* = 8) and resolved (*n* = 8) HCV subjects' T cell responses to rNS3, we measured recall responses in vitro (Table 1 and Figure 1). Responders were considered as those giving a response greater than 2 SDs above ${\overset{̅}{X}}_{\text{bkg}}$. Figure 1 shows the medium background subtracted from the triplicate response to give a Δ_cpm_ value plotted on a log scale, described in methods. As controls, we included H3 (3 *μ*g/ml), H5 (3 *μ*g/ml), and PHA (2 *μ*g/ml) (Figure 1). Each subject responded to PHA, and there was no statistically significant difference in the T cell response for any of the control antigens. However, rNS3 induced T cell proliferation in chronic subjects (3.1 SEM ± 0.17) was significantly attenuated (*P* < .05) compared to resolved subjects (4.01 SEM ± 0.15). Similarly, flow cytometric analysis of proliferating T cells stained with CFSE (Figure 2) reveals a significantly (*P* < .05) attenuated CD4^+^ T cell response in chronic HCV subjects (0.42 SEM ± 0.15) (*n* = 7) when compared to the resolved subjects (2.9 SEM ± 0.68) (*n* = 9) (Figure 2, Table 2). Although the CD8^+^ T cell response was not statistically significantly lower in the chronic compared to resolved T cell subjects, a trend towards lower CD8^+^ T cell response was evident in the chronic (1.8 SEM ± 1.8) compared to resolved (3.1 ± 0.7) (Figure 2, Table 2). 

**Figure 3:**
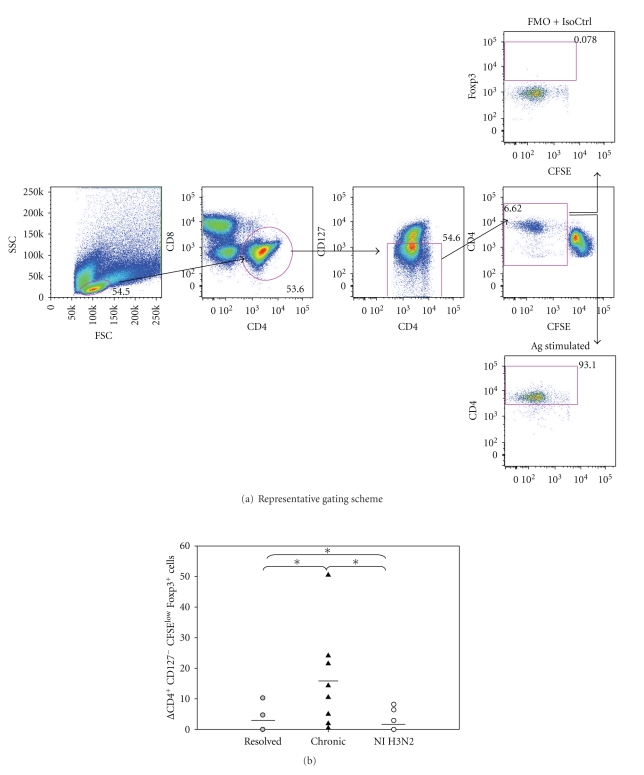
Higher frequency of Foxp3^+^ cells in chronic HCV subjects in comparison to resolved HCV subjects. (a) Back gating analysis of % Foxp3^+^ cells in CD4^+^ CD127^−^ CFSE^low^ cells. PBMCs were labeled with CFSE and then stimulated with either rNS3 (1 *μ*g/ml) or H3 (1 *μ*g/ml) and incubated for 7 days. The fluorescence minus one (FMO) plus isotype control for Foxp3 antibody was used to determine the gate for Foxp3^+^ cells (upper panel). An example of PBMC stimulated with an antigen is shown in the lower panel. (b) Chronic HCV patients (*n* = 9) have a significantly higher percentage (*P < .05 as determined by Student's *t*-test) of CD4^+^ CD127^−^ CFSE^low^ Foxp3^+^ expressing cells in comparison to resolved HCV subjects (*n* = 9), (Supplementary Table 2). Δ = [Experimental − Medium]. Noninfected subjects (*n* = 5) were stimulated with the recombinant H3 antigen as a comparison (Supplementary Table 2).

To determine if CD127 (IL-7R*α*) correlated with chronicity as determined by other laboratories [16], we used flow cytometric analyses to analyze the CD127 expression on both CD4^+^ and CD8^+^ T cells in chronic (*n* = 7), resolved (*n* = 10), and noninfected (NI) (*n* = 5) on Day 7 poststimulation antigen stimulation (see Table 1 in Supplementary Materials available online at doi:10.1155/2011/806061). Further, the upregulation of CD127 on CD4^+^ T cells has been shown to inversely correlate with Tregs in humans, and the negative selection of CD127 can be used as an accurate extracellular biomarker of Tregs as opposed to CD25 [15]. The noninfected subjects had a significantly (*P* < .05) lower expression of CD127^−^ (26 SEM ± 4.1) cells in comparison to resolved (40 SEM ± 2.8) and chronic (62.2 SEM ± 4.2) subjects (Supplementary Table 1) and the levels remained relatively consistent regardless of antigen stimulation. To test if HCV was able to induce antigen-specific Tregs, we analyzed CFSE dilution assays staining for CD4^+^ CD127^−^ CFSE^low^ cells at 7 days poststimulation (Figure 3(a)). Chronic HCV subjects (16.6 SEM ± 5.2) had a significantly higher expression of Foxp3 in antigen-specific CD4 T cells that were CD127^−^ (0 SEM ± 3.7) or negative controls stimulated with H3 (0 SEM ± 2.4) (Figure 3(b), Supplementary Table 2). These results suggest antigen stimulation with rNS3 causes expansion of regulatory T cells at a higher frequency in chronic HCV subjects compared to resolved HCV subjects.

### 3.2. Synthetic Peptide-Mix Experiments

**Figure 4:**
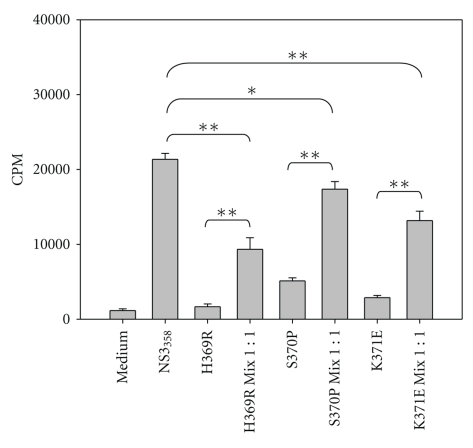
PBMC proliferative responses to NS3_358–375_ peptide variants. (a) HCV subject P.B3019 PBMC incubated for 3 h with peptide variants at 1 *μ*M then with the addition of 1 *μ*M wild-type peptide NS3_358–375_ where indicated. On Day 4, proliferating cells were labeled with 1 *μ*Ci ^3^H-TdR for the final 16 h of incubation, and cells were harvested for measurement of ^3^H-TdR incorporation on Day 5. Results are shown in mean counts per minute (CPM) +/- standard error of at least triplicate cultures. *P < .05 as determined by Student's *t*-test. **P < .005. Results are representative of greater than 10 experiments.

Previous work in our laboratory identified Th1 epitopes in a single HCV chronic subject and further characterized the viral variants that arose in one of the Th1 epitopes identified, NS3_358–375_ [21, 31]. Further, these variants were found not only to escape immune detection but were able to shift the cytokine profile from a Th1 cytokine pattern, which is correlated with viral clearance, to either a Th2 or Treg viral persisting response, respectively [21, 31]. Because variants and “wild-type” viruses exist together in the circulation, we attempted to simulate in vivo conditions by using what we have termed peptide-mixing experiments in cell-culture assays. Extensive work was performed using P.B3019 PBMCs to test the effect of various peptide concentrations and kinetics that each of the variants had on the cognate T-cell response (data not shown). It should be noted that our approach is very similar to previously described antagonism assays, with the exception being that we used polyclonal PBMCs instead of T-cell clones [32]. Our preliminary experiments showed that the proliferative response of PBMC preincubated with 1 *μ*M of variant peptide 3 hours prior to the addition of 1 *μ*M NS3_358–375_ peptide was inhibited (data not shown), which is consistent with an antagonism model. Further, if NS3_358–375_ peptide was added either before or at the same time as the variant peptide, there was no effect on NS3_358–375_ T cell proliferation (data not shown). Therefore, in subsequent experiments, variant peptide remained after the addition of the NS3_358–375_ peptide and cultures were incubated at 37°C at 5% CO_2_ for either 5 or 7 days then pulsed with ^3^H-Thymidine for the last 16–18 hrs. Cultures incubated with single peptide variants alone failed to stimulate as well as the wild-type NS3_358–375_ peptide (Figure 4). Further, peptide-mix cultures (variant(s) + wildtype) showed reduced levels of proliferation relative to those with NS3_358–375_ peptide alone (Figure 4).

### 3.3. Flow Cytometric Analysis of Inducible Tregs

**Figure 5:**
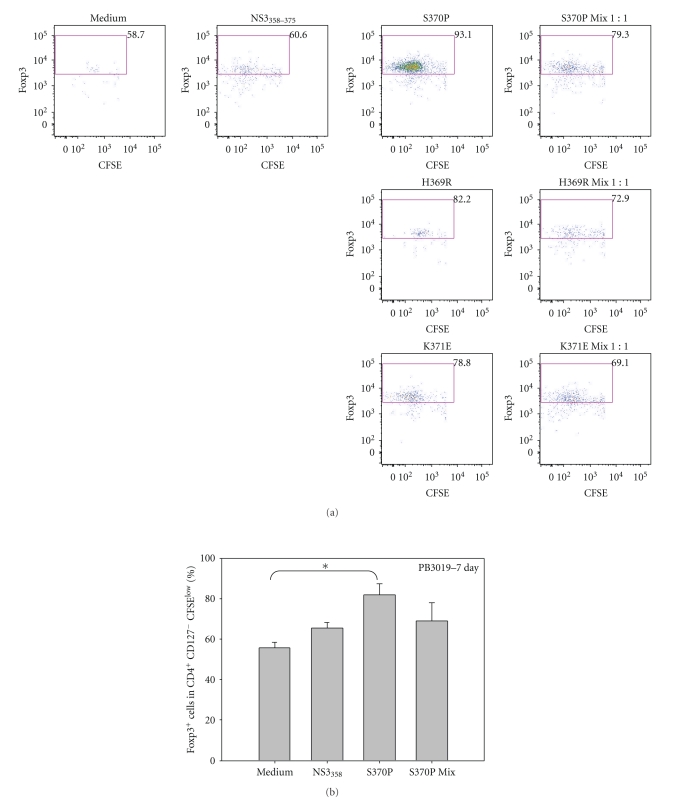
Increased Foxp3 expression in an antigen-specific manner by variant S370P stimulated PBMC from chronic P.B3019. (a) Representative flow plots of CFSE^low^ Foxp3^+^ cells from lymphocytes that were CD4^+^ CD127^−^ (gating scheme Figure 3(a)) stimulated with the indicated antigens at 1 *μ*M for 7 days. Peptide added to PBMC cultures is listed above the plot, and the percentages are the percent of CD4^+^CD127^−^CFSE^low^ cells. (b) Variant S370P $\left(\overset{̅}{x}=81.8\pm \text{SEM}\;5.7\right)$ stimulated PBMC significantly increases the expression of Foxp3 in CD4^+^CD127^−^CFSE^low^ cells above the medium background $\left(\overset{̅}{x}=55.73\pm \text{SEM}\;2.57\right)$ for P.B3019. *P < .05 as determined by Student's *t*-test.

To determine if variant S370P was able to induce Tregs in an antigen-specific manner, we stimulated P.B3019 PBMC with the indicated antigens, incubated the cells for 7 days, and analyzed the phenotype of proliferating cells in a CFSE dilution assay (Figure 3(a)). The induction of Foxp3 by variants H369R and K371E was not significantly higher in comparison to NS3_358_ peptide stimulated cultures but S370P induced a large population of Foxp3^+^ cells (Figure 5(a)). Multiple experiments were performed using S370P because S370P was the only variant that was stable for over 2 years in PB3019 (Figure 5(b)). PBMC from PB3019 (*n* = 3) increased Foxp3 expression (*P* < .05) when stimulated with S370P ($\overset{̅}{x}=\;81.8$%) (Figure 5(b)) in comparison to unstimulated culture ($\overset{̅}{x}=55.73$%) (Figure 5(b)).

### 3.4. CD4^+^CD25^+^ Treg Depletion

**Figure 6:**
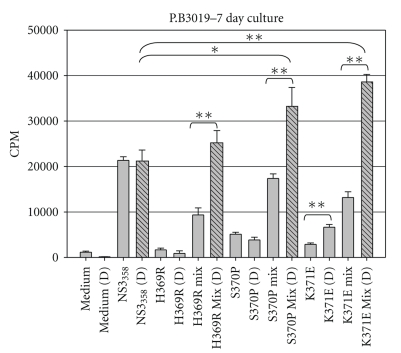
PBMC proliferative responses to NS3_358–375_ peptide variants and mixes with wild-type peptide in cultures depleted of CD4^+^CD25^+^ Treg cells. Subject 3019 total or Treg-depleted PBMC were stimulated with peptide variants and measured ^3^H-TdR-uptake. Results are mean counts of six wells each +/- SEM, representative of three independent experiments. Gray bar, individual peptide; stripped bars, CD4^+^CD25^+^ Treg-depleted PMBC. *P < .05 as determined by Student's *t*-test. **P < .005.

To determine if naturally occurring variants inhibit T cell proliferation, we used commercially available CD4^+^CD25^+^ regulatory T cell isolation kit to deplete CD4^+^CD25^+^ T cells from a PBMC pool prior to stimulation with the NS3_358–375_ and variant peptides (Figure 6). Depletion of CD4^+^CD25^+^ Tregs enhanced the proliferation of T cells in response to NS3_358–375_ peptide (Figure 6, gray versus black stripped bars, resp.). Furthermore, stimulation with the K371E variant alone in cultures depleted of CD4^+^CD25^+^ Treg cells led to an increase in the proliferation level, suggesting that at least one mechanism by which the peptide variants suppress effector T cell responses is through the induction of Tregs.

### 3.5. MHC Class II Tetramer Staining Using Multiple HCV Subjects

**Figure 7:**
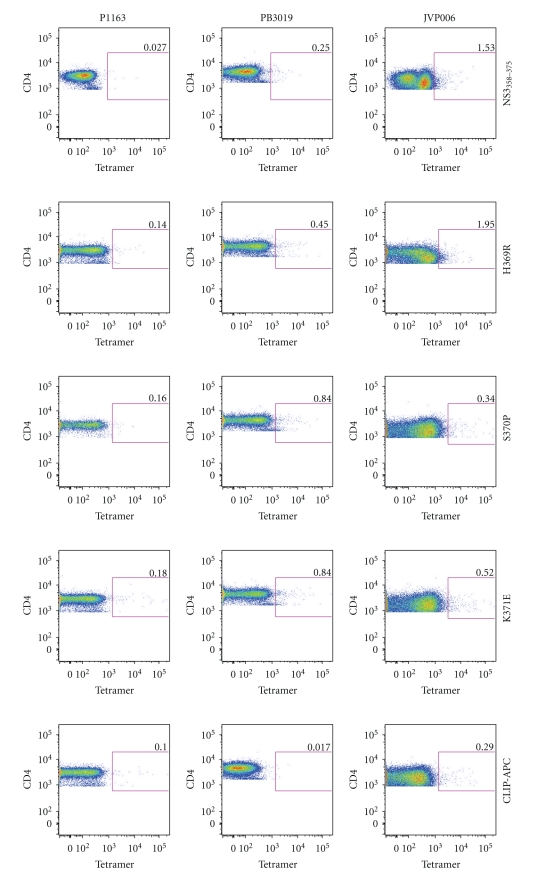

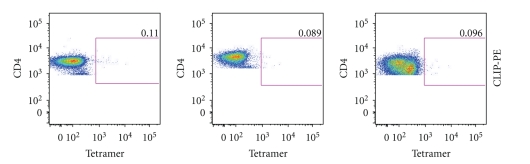
DRB1*1501 MHC class II variant and cognate tetramers are able to bind to CD4^+^ T-cells from multiple patients. PBMCs from a noninfected (P1163), chronic (P.B3019), and resolved (JVP008) were individually stained with DR15 MHC class II tetramers (2 *μ*g/ml). P.1163 is a noninfected DR15 subject and was used as a control to test for nonspecific labeling of each DR15 tetramer (first column). DR15-CLIP-APC and -PE tetramers were used as a control for each experiment (bottom two rows).

To determine if DR15 MHC class II tetramers loaded with NS3_358–375_, or the variant peptides H369R, S370P, and K371E, were able to bind to CD4^+^ T cells, PBMCs from multiple HCV subjects were stained for antigen-specific T cells (Figure 7). P.1163, a noninfected DR15 subject (Table 1), was used as a control. Also, DR15 CLIP-PE and -APC tetramers were used as controls in each experiment (Figure 7, bottom two rows). Both P.B3019 (chronic infection) and JVP008 (resolved infection) had detectable DR15-restriced CD4^+^ T cells with varying avidities for the tetramers (Figure 7). Finding HCV-specific T cells in more than one HCV subject suggests that this is not an idiosyncratic phenomenon although larger numbers of patients need to be studied in order to determine whether our results reflect a more general observation.

### 3.6. MHC Class II Tetramer Depletion Assays

**Figure 8:**
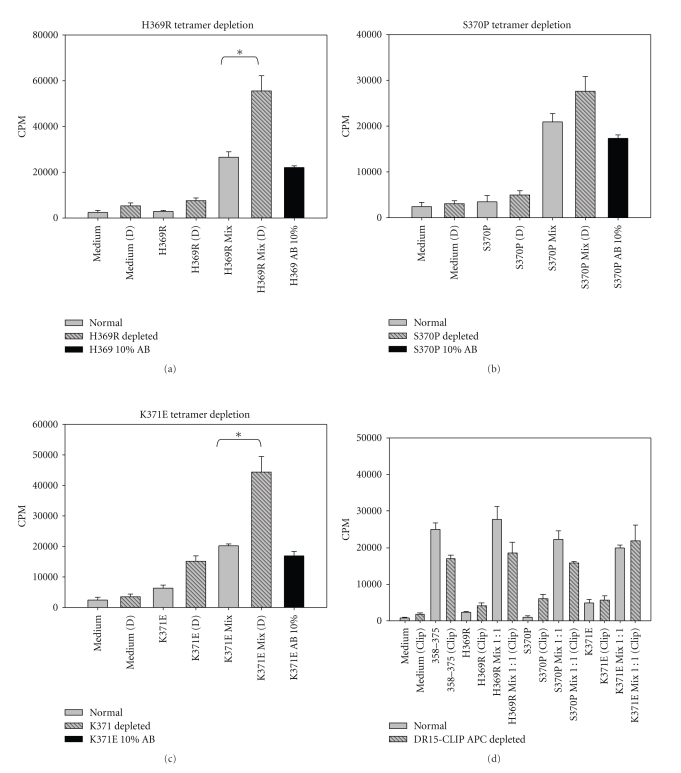
Tetramer depletion of T cells specific for variant peptides restores T cell proliferative response to cognate NS3_358–375_. (a–c) Variant tetramer depleted listed at the top of the panels. Tetramer depletion was done using PBMC from subject PB3019. PBMC incubated with variant peptide for 3 hrs and then stimulated with WT peptide for 7 days (gray, stripped, and black panels). Black histogram is indicative of add-back experiment, in which, 10% represents approximately the same number of cells as normal mix population. Each experiment was done in triplicate, replicated 3 times for a total 9 data points. (d) HLA-DR15 CLIP tetramer-depleted cells shown in yellow compared to PBMC from nondepleted PBMC proliferation assay at 7 day, each experiment was done in triplicate (*n* = 2).

To test the specificity of variant-induced suppression, PBMCs were stained with variant-loaded tetramers, and magnetic beads were used to remove tetramer-positive T cells; such depleted PBMC cultures were subsequently stimulated with peptides as indicated (Figures 8(a)–8(d)). Both H369R and K371E tetramer-depleted cultures responded better to NS3_358–375_ peptide in comparison to the nondepleted cultures (Figures 8(a), 8(c)). Although S370P tetramer depleted cultures did not have a statistically significantly higher T cell response in comparison to nondepleted cultures, S370P-depleted cultures consistently showed an enhanced T cell response to NS3_358–375_ (Figure 8(b)). Adding-back tetramer positive cells to depleted cultures restored suppression (Figures 8(a)–8(c), black bars). Using a nonspecific control tetramer showed no effect on T cell responses to the NS3_358–375_ peptide (Figure 8(d)). The results indicate that tetramer depletion of variant-specific T cells enhances T cell proliferative response to the NS3_358–375_ cognate peptide in an antigen specific manner.

### 3.7. MHC Class II Tetramer Add-Back Assays

**Figure 9:**
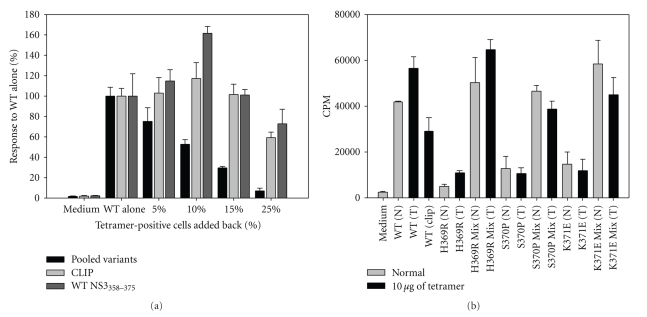
Tetramer-positive cells suppress T cell proliferation in a dose-dependent manner. P.B3019 PBMCs were stained with pooled variants of H369R, S370P, and K371E (black), CLIP (light gray), or WT NS3_358–375_ (dark gray) and removed by bead depletion. After tetramer depletion, the cultures were stimulated with NS3_358–375_ peptide. Tetramer-positive cells were volumetrically added back into culture with P.B3019 NS3_358–375_ stimulated PBMCs. Data is representative of percent response to NS3_358–375_ stimulated PBMCs depleted with indicated tetramer(s) (WT alone) and set to 100%. Controls were CLIP (light gray) and NS3_358–375_ (dark gray). (b) PB3019 PBMC cultures were stimulated with indicated peptide(s) with each tetramer added at 10 *μ*g/ml and incubated for 7 days.

To address the potency of variant-specific T cells to suppress the cognate T cell response, we added variant tetramer positive cells back into PBMC culture stimulated with NS3_358–375_ peptide. After tetramer depletion, PBMC cultures were stimulated with NS3_358–375_ peptide (Figure 9(a)). Because of variability in levels of proliferation, responses in the presence of each variant were normalized to WT alone; note the effect of dilution upon responsiveness at 25% (vol/vol). T cell responses in the restored presence of all variant-specific T-cells were suppressed in a dose-dependent manner. To control for nonspecific depletion and suppression, we used a CLIP-loaded tetramer, which resulted in no effect on the T cell response. Depletion of PBMC cultures with NS3_358–375_ tetramer and subsequent add-back of NS3_358–375_-specific T cells actually enhanced proliferation at lower volumes. This result is not due to toxicity because the same concentration of tetramer (10 *μ*g/ml) added into culture along with the respective peptide(s) at 1 *μ*M resulted in no inhibition of T cell proliferation (Figure 9(b)). We conclude from these experiments that variant-tetramer-positive T-cells are able to suppress T cell proliferation to wild type NS3_358–375_ *in vitro*. To our knowledge this is the first demonstration of what may be antigen-specific regulatory T cells.

### 3.8. MHC Class II Tetramer Staining of NS3_358–375_-Specific CD4^+^ T Cells

**Figure 10:**
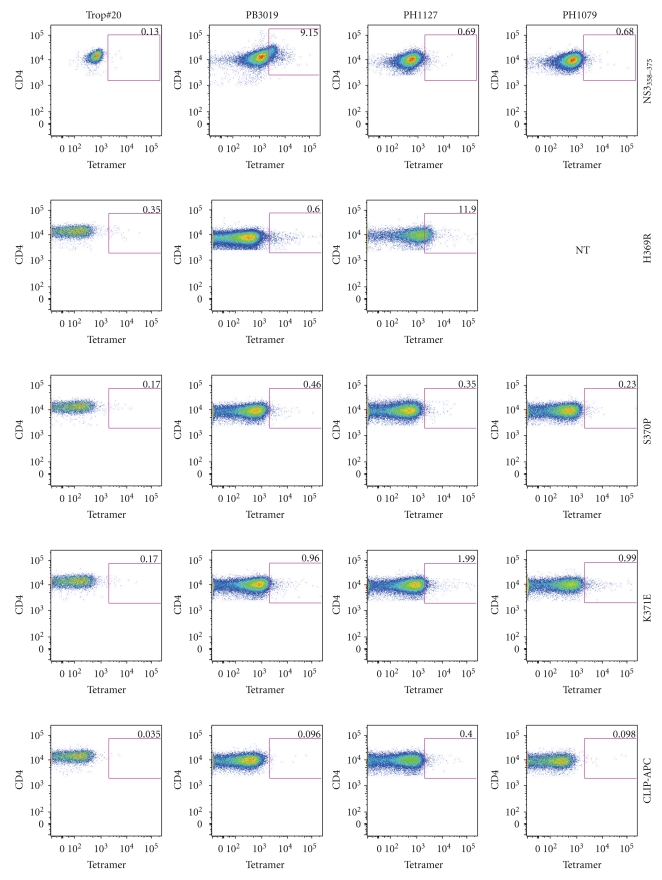

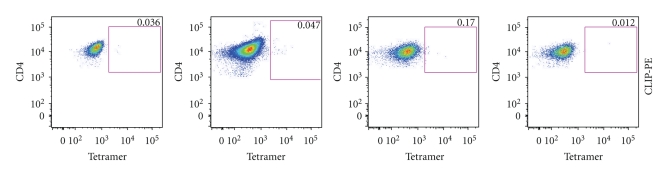
MHC class II tetramers are able to stain NS3_358–375 _antigen-specific CD4^+^ T cells. PBMC from PB3019, PH1127, and PH1079 were prestained with CFSE and stimulated with NS3_358–375_ synthetic peptide for 7 days. The CFSE^low^ cells were sorted and then stained with each tetramer. The cells are >99% CD4^+^ as determined by flow cytometry (data not shown). Trop no. 20 are CFSE^low^ tropomysoin-specific CD4^+^ T cells expanded by the same procedure as NS3_358–375_. Trop #20 was used to control for tetramer specificity (first column). Nonspecific tetramer (CLIP) was used for each experiment (bottom 2 panels). NS3_358_ and variant MHC class II tetramers stain PB3019, PH1127, and PH1079 CD4^+^ CFSE^low^ T cells. Not Tested (NT). Tetramer used is labeled on the right.

To extend our observation that variant-specific T cells are able to bind to cells that are specific for the cognate peptide in a somewhat larger cohort of subjects, we amplified CD4^+^ T-cells specific for NS3_358–375_ peptide. PBMCs from HLA-DR15 subjects (Table 1) were prelabeled with (0.5 *μ*M) CFSE and stimulated with NS3_358–375_ synthetic peptide for 7 days. The CD4^+^ CFSE^low^ cells were sorted, expanded with CD3/CD28 beads, and stained with tetramer following removal of beads. All cultures were >99% CD4^+^ as determined by flow cytometry (data not shown). Trop no. 20 (HLA-DR1/3) are CFSE^low^ CD4^+^ T cells expanded in the same manner except that shrimp tropomyosin was substituted for NS3_358–375_ and served as an additional negative control (Figure 10, first column). Nonspecific tetramer (CLIP)-APC and -PE were also used as controls in each experiment (Figure 10). As might be expected, NS3_358_ and variant MHC class II tetramers stain PB3019, PH1127, and PH1079 CD4^+^ antigen-specific T cells all of which share or require the HLA-DR15 restriction element. Thus, tetramers loaded with either cognate or variant peptides are likely to bind overlapping subsets of T-cells found in PBMC from multiple subjects.

## 4. Discussion

We demonstrate in vitro induction of regulatory T cells capable of suppressing antigen-specific T cell responses. We postulated that previously defined viral variants in a Th1 epitope could be responsible for the induction of Tregs based on the cytokine shift and attenuated T cell response [17]. Further, chronically infected subjects exhibited significantly lower T cell responses in comparison to resolved subjects. These attenuated T cell responses correlated with the induction of the Treg lineage-specific markers in proliferating T cells specific for rNS3. 

Although Fuller et al. [33] identified and tracked HCV NS3 viral variants in MHC class II-restricted epitopes in an infected chimpanzees, similar to our previous work [20], but it was not clear if the viral variants were able to affect the T cell response to cognate peptide. Our study demonstrated that viral variants attenuated T cell responses to cognate peptide and not unrelated peptide. Further, the specific variant, S370P, induced Foxp3 in an antigen-specific manner in a chronic HCV patient. In an effort to generalize our finding from one chronic subject, we were able to detect variant-specific T cells in multiple HLA-matched subjects. The ability to detect the wild-type positive T cells along with variant-specific T cells suggests that the mechanism of Treg induction by naturally occurring epitope variant is likely not exclusive to one chronically infected individual, albeit the functional studies, though cumbersome, now need to be done in a larger cohort of chronic and resolved HCV patients. 

Previous studies had found that depletion of CD4^+^CD25^+^ cells enhanced HCV-specific T cell response to HCV antigens; however, these studies also described enhanced T cell response to control antigens from Epstein-Bar Virus (EBV), Cytomegalovirus (CMV), and influenza indicating that the depletion of this subset of cells is not specific for HCV [4, 34]. Depletion of CD4^+^CD25^+^ cells restored PBMC proliferative responses to NS3_358–375_ to levels that matched or exceeded those in the nondepleted PBMC cultures that were preincubated with variant peptide. An increase in the level of proliferation induced by variant K371E alone following CD4^+^CD25^+^ cell depletion implied a suppressive role for CD4^+^CD25^+^ Treg cells. These results suggest that nonspecific depletion of Tregs enhanced T cell proliferation.

Compelling evidence for HCV-specific Tregs by Ebinuma et al. [35] identified CD4^+^CD25^+^ Foxp3^+^ MHC class II tetramer positive cells in peripheral blood of HCV patients. Further, Heeg et al. [7] performed a longitudinal study using MHC class II tetramer staining to track HCV-specific CD4^+^ Foxp3^+^ T cells during the course of HCV infection in a cohort of patients. Although Heeg's study did not find a correlation between an increase in Foxp3 expression and viremia, they did observe an attenuated antigen-specific T cell proliferative response and lowered IFN*γ* secretion in MHC class II tetramer positive cells. These studies did not identify viral variants arising in the epitopes analyzed, therefore, giving no indication if viral variants could have an effect on the cognate T cell response [7, 35]. Depleting HCV-specific T cells that bind to MHC class II variant tetramers, we found an enhanced T cell proliferative response to NS3_358–375_ peptide and a restoration of suppression when the variant-specific T cells were added back. Although the depletion of S370P tetramer positive cells did not significantly enhance the proliferative response over the nondepleted culture, the variant tetramer depletion results suggest that the avidity of the tetramers for NS3_358–375_-T cells are different, leading us to hypothesize that these variants could be acting as altered peptide ligands. Consistent with this, K371E had a higher T cell response when CD4^+^CD25^+^ cells and K371E tetramer positive cells were removed indicating that this variant might affect yet a different subset of cells. Further, adding variant tetramer positive cells back into NS3_358–375_ stimulated cultures had a dose-dependent suppressive effect, suggestive of Tregs. Taken together, our results suggest antigen-specific Tregs are responsible for suppression of an effector T cell response, and we believe that a possible mechanism for this phenomenon is that Hepatitis C viral variants may act as APLs to induce Tregs. Previous [21, 36] and current work in our laboratory clearly demonstrates that viral variants are able to antagonize cloned T cells specific for NS3_358–375_. Further, the variant peptides loaded onto MHC class II have different avidities for T cells that are specific for NS3_358–375_, which suggests that these variants are acting as naturally occurring altered peptide ligands (paper in preparation).

It has been observed that wild-type HCV sequences remain stable in humans and chimpanzees even years into an ongoing infection [2, 31, 33, 37]. We have shown that approximately 80% of the circulating virus has “wild-type” 1A sequence [21, 31]. Interestingly, the S370P variant was found in two isolates separated by 2 years, the variation has not impacted viral fitness negatively, yet its frequency seems not to have increased with time as might be expected with other escape models. Indeed, of all variants within the NS3_358–375_ epitope tested to date, none has lost the ability to bind the DR15 class II restriction element, which is contrary to a classic evasion escape model [38]. The fact that HCV epitope variants seem to induce a functional unresponsiveness in peripheral T cells implies a radically different viral strategy as well host-related immunopathogenesis. HCV seems to have developed the ability to induce a specific tolerance to itself by exploiting natural mechanisms that operate within the host. Our data suggest the hypothesis that viral mutation leads to APL that blunts specific helper T cell responses, which thereby attenuates the usual effector mechanisms requisite for antibody and killer T cell induction.

## 5. Conclusion

In conclusion, we have shown for the first time that variants of an HCV immunodominant epitope, which arose during chronic infection in a human, induced Foxp3 expression in an antigen-specific manner and had a dose-dependent suppressive effect in vitro, perhaps reflective of regulatory T cells. While the number of individuals studied to this point is small, we know that such variation occurs in other individuals and applies to the cytotoxic effector arm of the immune response as well [39]; notably, this latter study was performed in an HLA-DR15-positive subject. Therefore, our in vitro results imply that selective immune driven viral variants do not “escape” immune detection, similar to observations by Fuller et al. [33], but rather they avoid the consequences of immune recognition by inducing antigen-specific Tregs which in turn provide “immunological cover” for wild-type viral sequences including those that contain the NS3_358–375_ epitope, which should otherwise be recognized, provide effective T cell help such that virus can be appropriately eliminated. Our results do not oppose other mechanisms for viral persistence, but may act in concert to subvert the adaptive immune response to HCV.

## Supplementary Material

Supplementary Tables 1 and 2 provide each individual subject's frequency of indicated biomarkers.

## Figures and Tables

**Table 1:** HCV and HLA genotypes of HCV subjects. HCV and HLA types of chronic and resolved subjects used in this study. All subject's PBMC were incubated with recombinant NS3 protein (rNS3) in a dose-dependent manner using a proliferation assay to detect T cell responses. All subjects were screened for HCV RNA by quantitative PCR. In the case of resolved subjects, in which they had no detectable viral load, a RIBA was performed to screen for HCV antibodies. *subjects used for T cell proliferation assay in Figure 1.

	Subject ID	HCV status	Genotype	HLA-DR	AB
∗	RLM 037	R	−	1,15	+
∗	KML044	R	−	9,15	+
∗	ZSS035	R	−	1,15	+
∗	LEC028	R	−	1,8	+
∗	KTJ010	R	−	1,15	+
∗	BPB026	R	−	3,15	+
∗	DRB012	R	−	7,7	+
∗	JVP008	R	−	15,15	+
	JPZ061	R	−	4,4	+
	PH1127	R	−	13,15	+
	PH1079	R	−	4,15	+
∗	AJG066	C	1A	1,8	ND
∗	KRW002	C	1A	8,14	ND
∗	MH065	C	1A	14,15	ND
∗	P.B3019	C	1A	15,7	ND
∗	NLM049	C	1A	11,13	ND
∗	CER014	C	1A	7,13	ND
∗	DRB051	C	1A	1,13	ND
∗	SSB007	C	1A	7,13	ND
	RLW027	C	1A	4,15	ND
	P.1022	NI	−	8,10	−
	P.1163	NI	−	1,15	−
	P.1078	NI	−	9,13	−
	P.1127	NI	−	5,6	−

**Table 2:** CD4^+^ and CD8^+^ T cell responses to rNS3 and H3 Ags as shown as frequency of CFSE^low^ and the transformation of T cell proliferation data. Frequency of CD4^+^ and CD8^+^ T cells in CFSE-labeled lymphocytes when stimulated with rNS3 and H3 at 1 *μ*g/ml for 7 days and analyzed by flow cytometry. On Day 7, the cells were stained with CD4 and CD8. The proliferative response was determined by the medium for each subject. log_10_Δ_max_ is the transformation of the T cell prolfieration data for each subject and the respective antigen stimulation.

Subject		Medium		NS3			H3		
		CD4	CD8	CD4	CD8	log_10_Δ_max _	CD4	CD8	log_10_Δ_max _
RLM 037	R	0.97	1.49	6.97	7.1	4.4	1.49	4.91	0
KML044	R	0.29	0.53	4.06	10.7	4	0.27	0.63	0
ZSS035	R	0.6	0.95	4.12	2.65	4.2	3.73	1.85	4.1
LEC028	R	1.16	0.54	6	2.4	4	1.81	0.8	2
KTJ010	R	1.33	1.32	4.98	2.92	4	1.88	1.95	2.5
BPB026	R	0.4	0.29	1.58	1.12	3.2	0.42	0.22	2.7
DRB012	R	1.22	0.77	0.8	3.68	2.5	0.8	3.68	3.5
JVP008	R	0.4	0.46	2.61	4.07	3.2	0.8	0.47	3
JPZ061	R	1.34	1.76	1.1	1.41	3.3	1.15	1.3	0
AJG066	C	4.31	4.84	4.73	4.8	3.4	4.81	4.68	0
KRW002	C	4.79	4.6	5.29	5.19	3.8	5.43	4.75	3.5
MH065	C	6.31	7.21	6.97	7.29	3.6	6.6	7.3	3.2
NLM049	C	1.84	4.47	2.01	14.2	3.5	0.18	0.84	3.05
CER014	C	0.68	0.34	1.04	0.75	4	0.74	0.33	3
SSB007	C	22.7	29.1	23.8	31.5	3.9	23.1	28.1	0
RLW027	C	0.16	0.44	0.33	1.99	4.2	0.18	0.84	3.8
