# Supplementary material for: Hepatitis C Virus Induces Regulatory T Cells by Naturally Occurring Viral Variants to Suppress T Cell Responses
Source: Clin Dev Immunol. 2010 Dec 6;2011:806061. doi: 10.1155/2011/806061 (PMC3004422; doi:10.1155/2011/806061)
Supplement: Supplementary file 1 — Supplementary Tables 1 and 2 provide each individual subject's frequency of indicated biomarkers. [file 806061.f1.pdf]

## Supplementary Table 1. CD4$^+$ and CD8$^+$ CD127$^-$ expression

| | | CD127- | | | | | |
|---|---|---|---|---|---|---|---|
| | | Medium | | NS3 | | H3 | |
| | | CD4 | CD8 | CD4 | CD8 | CD4 | CD8 |
| Subject | | | | | | | |
| RLM 037 | R | 60.1 | 28 | 65.1 | 32.9 | 62.1 | 28.9 |
| KML044 | R | 37.6 | 23.6 | 46.4 | 29.7 | 42.3 | 24.7 |
| ZSS035 | R | 38.7 | 14.1 | 26.2 | 9.72 | 42.5 | 17.9 |
| DOY041 | R | 40.7 | 20.3 | 42.7 | 18.1 | 46.1 | 24.3 |
| LEC028 | R | 48.7 | 30.8 | 54.6 | 26.8 | 51.8 | 34.7 |
| KTJ010 | R | 28.7 | 8.1 | 38.5 | 11 | 26.4 | 7.62 |
| BPB026 | R | 32.8 | 22 | 37.1 | 9.75 | 33.1 | 10.5 |
| DRB012 | R | 41 | 17.1 | 47.4 | 19.7 | 46.9 | 19 |
| JVP008 | R | 35.9 | 15.4 | 42.4 | 19.7 | 34.2 | 16.2 |
| JPZ061 | R | 38.6 | 18.7 | 44.6 | 27.9 | 45.5 | 27.3 |
| AJG066 | C | 84 | 62.8 | 84 | 63.3 | 83.2 | 60.7 |
| KRW002 | C | 62.8 | 54.7 | 63.9 | 57.9 | 64.1 | 57.5 |
| MH065 | C | 51 | 54.7 | 44.7 | 45.5 | 51.7 | 56.8 |
| NLM049 | C | 60.6 | 35.9 | 54.1 | 37.1 | 63.1 | 38.6 |
| CER014 | C | 66.2 | 27.2 | 61 | 23.6 | 67 | 29.8 |
| SSB007 | C | 59.2 | 33.3 | 52.9 | 23.8 | 61.1 | 35.9 |
| RLW027 | C | 51.8 | 56.1 | 52.3 | 53.5 | 58.4 | 68 |
| P.1022 | NI | 12.2 | 10.3 | 40.4 | 30.1 | 26.2 | 18.4 |
| P.1163 | NI | 37.2 | 14 | 34.4 | 15.3 | 33.7 | 13.5 |
| P.1078 | NI | 25.3 | 23.9 | 28.4 | 23.5 | 27 | 26.3 |
| P.1127 | NI | 31 | 20.1 | 24.6 | 12.1 | 25.1 | 13.7 |
| P.1031 | NI | 27.6 | 23.7 | 35.7 | 12.7 | 34.8 | 23.2 |

Frequency of CD4$^+$ CD127$^-$ and CD8$^+$ T CD127$^-$ cells in lymphocyte population when stimulated with rNS3 and H3 at 1μg/ml for days and analyzed by flow cytometry.

## Supplementary Table 2. CD4+ CD127- CFSElow Foxp3

| Subject | | Medium | NS3 | H3 |
|---|---|---|---|---|
| | | $CD4^+$ $CD127^-$ $CFSE^{low}$ $Foxp3^+$ | | |
| RLM 037 | R | 83.1 | 66 | 68.4 |
| KML044 | R | 86.7 | 62 | 72.3 |
| ZSS035 | R | 82.6 | 71.8 | 81.8 |
| LEC028 | R | 79.3 | 84 | 85 |
| KTJ010 | R | 47.4 | 57.7 | 41.9 |
| BPB026 | R | 30.9 | 25 | 14.3 |
| DRB012 | R | 61.4 | 46.4 | 46.4 |
| JVP008 | R | 15.9 | 14.1 | 29.7 |
| JPZ061 | R | 36.9 | 26.1 | 20.4 |
| AJG066 | C | 52.3 | 66.6 | 57.2 |
| KRW002 | C | 10.4 | 12.3 | 10.1 |
| MH065 | C | 18.6 | 40.1 | 37.7 |
| P.B3019 | C | 58.7 | 69.2 | 71.5 |
| NLM049 | C | 16.5 | 16.9 | 31.7 |
| CER014 | C | 18.6 | 40.1 | 37.7 |
| DRB051 | C | 16.5 | 67 | 49.5 |
| SSB007 | C | 12.3 | 17.3 | 10.9 |
| RLW027 | C | 16.7 | 40.8 | 20 |
| P.1022 | NI | 0 | 26.8 | 8.2 |
| P.1163 | NI | 18.1 | 28 | 21 |
| P.1078 | NI | 14.1 | 5.178 | 8.68 |
| P.1070 | NI | 18.6 | 16.3 | 11.8 |
| P.1031 | NI | 16 | 0 | 7.69 |

Frequency of $CD4^+$ $CD127^-$ $CFSE^{low}$ $Foxp3^+$ cells in lymphocyte population when stimulated with rNS3 and H3 at 1μg/ml for days and analyzed by flow cytometry.
